# More Doctors, Less Competency? A Quality Crisis in Brazilian Medical Education

**DOI:** 10.1111/jep.70436

**Published:** 2026-04-08

**Authors:** Bráulio Evangelista de Lima, João Victor Rosa de Freitas, Fernanda Patti Nakamoto, Paulo Sérgio Sucasas da Costa, Núbia de Souza Lobato, Claudio Andre Barbosa de Lira

**Affiliations:** ^1^ Institute of Health Sciences Federal University of Jataí Jataí Goiás Brazil; ^2^ Postgraduate Program in Health Sciences, Faculty of Medicine Federal University of Goiás Goiânia Goiás Brazil; ^3^ Faculty of Physical Education and Dance Federal University of Goiás Goiânia Goiás Brazil; ^4^ São Camilo University Center Ipiranga São Paulo Brazil

Since 2004, Brazil has more than tripled the number of medical schools, increasing from 143 to 448 by 2024, while annual seats rose from 13,820 to 48,491 over the same period [1]. Expanding medical training capacity is a legitimate response in a continental country facing persistent physician shortages and marked geographic maldistribution, particularly in underserved regions. However, this rapid expansion has not been accompanied by proportional growth in residency training capacity, limiting opportunities for supervised transition to practice and increasing reliance on undergraduate training quality [1].

Although Brazil has a sophisticated official system for educational assessment, with a strong emphasis on institutional infrastructure, regulatory compliance and programme‐level characteristics [2], student performance has received comparatively less emphasis. However, there is no standardized mechanism for systematically evaluating clinical competency. Furthermore, many newly established institutions lack integration with student‐led clinics and hospitals, provide insufficient clinical training settings and have constrained faculty development structures [3]. Against this regulatory backdrop, the unprecedented scale and speed of medical school expansion have reignited long‐standing concerns about the quality of medical education, particularly regarding minimum competency at graduation and the training of future physicians. These concerns have been brought into sharp focus by the release of the first results of the National Examination for the Evaluation of Medical Training (ENAMED). Similar examinations have been implemented in several countries as mechanisms to ensure minimum competence in medical practice. Evidence from other contexts suggests that performance in such examinations may be associated with aspects of subsequent physician performance and patient outcomes, supporting their role as components of broader quality assurance systems in medical education [4, 5].

In this commentary, we argue that the early ENAMED results should be interpreted not merely as isolated examination outcomes, but as a warning signal that Brazil's rapid expansion in medical training capacity may be outpacing its ability to assure minimum competency at graduation, with implications for patient safety, equity and health‐system planning.

ENAMED is a Brazilian national examination created by the Ministry of Education, and it was introduced in 2025, with plans to be administered annually to all graduating medical students [6, 7]. The primary objective of the examination is to assess whether graduating medical students have acquired the competencies and skills required by the Brazilian medical education government legislation [8]. In addition, the examination aims to: (i) generate evidence to support improvements in undergraduate medical education and strengthen the quality of medical training in Brazil; (ii) serve as a pathway for admission to medical residency programmes; (iii) help ensure that future physicians are adequately prepared to practice within the Brazilian Unified Health System (Sistema Único de Saúde – SUS); and (iv) establish a unified national instrument for evaluating the quality of medical training in the country.

In its inaugural edition, the examination consisted of 100 multiple‐choice questions with an interdisciplinary and multiprofessional orientation, emphasizing problem‐solving and the assessment of theoretical knowledge, clinical reasoning and essential competencies across increasing levels of complexity [9]. The examination items were developed by faculty members from higher education institutions involved in undergraduate medical education who were selected through a public national call. All selected item writers held medical degrees and specialist qualifications in at least one of the following core areas of medicine: internal medicine, general surgery, paediatrics, obstetrics and gynaecologist, family and community medicine, public health or mental health.

ENAMED is administered by the National Institute for Educational Studies and Research (INEP) in collaboration with the Brazilian Hospital Services Company (EBSERH). INEP is a federal agency linked to the Ministry of Education whose mission is to produce national education statistics, conduct research and implement large‐scale assessments to monitor the quality of the Brazilian education system and inform public policy [10]. EBSERH is a federal public company that manages student‐led hospitals affiliated with federal universities, supporting medical education, research and the provision of healthcare services through the SUS [11].

Despite the existence of a Brazilian medical education government legislation defining expected competencies, recent ENAMED results suggest that achieving consistent competency outcomes across institutions remains challenging. Indeed, the results of the first edition are troubling. Among the 351 medical schools evaluated, 107 (30.4%) received unsatisfactory ratings (concepts 1–2 on a scale from 1 [lowest] to 5 [highest]), and a further 80 (22.8%) achieved only the minimum acceptable level (concept 3). Among 39,258 graduating students, over 13,000 (33.1%) individuals failed to reach the minimum proficiency standard defined by the exam. More concerning, these individuals have already graduated and are entering the medical workforce [12, 13].

Evidence on high‐stakes national examinations shows they can serve as independent safeguards, predict aspects of future physician performance and patient outcomes, particularly when workplace assessment suffers from leniency or ‘failure to fail’ [14]. Thus, ENAMED represents a significant shift in Brazilian medical education policy by introducing a standardized exam and a nationwide benchmark of minimum competency. Therefore, the exam should not be viewed as punitive, but as a diagnostic tool capable of identifying systemic weaknesses in undergraduate training that institutional self‐assessment may miss.

At the same time, high‐stakes national examinations are not free of unintended consequences. For example, high‐stakes examinations such as ENAMED may divert time and resources towards test preparation, increase learner stress and risk reproducing inequities when examination readiness is shaped by structural disparities between institutions [15, 16]. International debates on national licensing examinations show that, while such assessments may contribute to standardization and may correlate with later performance outcomes, the broader evidence that they independently improve practice standards or patient safety remains limited [15, 16]. Differences in examination performance across institutions may reflect a range of factors, including variations in curriculum design, governance of educational programmes, student selection processes and clinical training environments.

Importantly, within the Brazilian context, where a substantial proportion of medical schools are privately operated, policy responses should prioritize strengthening accreditation standards and regulatory oversight to ensure minimum training conditions across institutions. In this sense, examination results should be interpreted not only as indicators of student performance but also as signals for improving governance and quality assurance within the medical education system. For this reason, ENAMED should be understood as one component of a broader quality‐assurance strategy rather than as a stand‐alone proxy for educational quality.

Although the results of a single examination should be interpreted with caution, the ENAMED findings raise significant concerns. Notably, students with the highest scores are most likely to secure residency positions, where clinical skills are further developed under supervision. In a context where the number of undergraduate medical admissions far exceeds the availability of residency positions, it is reasonable to assume that lower‐performing graduates will face substantial barriers to accessing postgraduate training. As a result, a segment of newly qualified physicians may enter independent practice with limited opportunities for structured clinical improvement.

These risks are unlikely to be evenly distributed across the health system. Structural factors in workforce allocation mean that underserved and resource‐constrained regions often experience lower availability of supervision and support, potentially amplifying the consequences of variability in baseline training. In such contexts, insufficient baseline competency may exacerbate existing inequities in access to safe and effective care, disproportionately affecting populations already exposed to structural vulnerabilities. Therefore, ensuring educational quality at the point of graduation is not only an academic concern but a critical component of equity‐oriented health system governance, underscoring the importance of the training provided by Brazilian medical schools.

Altogether, these findings point to a potentially consequential gap in baseline competency at medical graduation. If left unaddressed, this gap risks increasing preventable harm, eroding public trust in the medical profession and further straining an already overburdened health system.

Facing this challenge will require coordinated action across multiple levels. First, expansion of undergraduate medical training should be more closely aligned with residency capacity to ensure adequate opportunities for supervised clinical development after graduation. Second, national regulatory bodies should strengthen minimum requirements for clinical training infrastructure, including access to student‐led hospitals, structured supervision and qualified faculty development programmes. Third, accreditation and regulatory oversight could incorporate national assessment data, such as ENAMED results, as one component of broader quality assurance systems while avoiding overreliance on a single metric. In addition, targeted support and remediation pathways should be developed for graduates who do not initially meet minimum competency thresholds. Finally, policies should address institutional heterogeneity by strengthening oversight of educational governance and ensuring that the rapid expansion of medical education is accompanied by robust mechanisms to safeguard training quality.

It is worth mentioning that our argument is limited by reliance on early results from the first edition of ENAMED, without longitudinal follow‐up linking examination performance to residency access, clinical performance or patient outcomes. Future research should examine whether ENAMED predicts downstream practice quality and whether its implementation narrows or widens educational and institutional inequities across regions.

From a broader perspective, the Brazilian experience offers a policy‐relevant signal for health systems globally: rapid expansion of medical schools without robust mechanisms to monitor, support minimum competency and ensure a safe transition to practice may undermine rather than advance the goals of public health and health equity.

## Author Contributions

All authors contributed equally to the conception and design of the commentary, as well as the drafting and reviewing of the intellectual content and approved the commentary in its final form.

## Ethics Statement

The authors have nothing to report.

## Conflicts of Interest

The authors declare no conflicts of interest.

## Data Availability

Data sharing is not applicable to this article as no data sets were generated or analysed during the current study.
